# Repeat Cytoreduction and Hyperthermic Intraperitoneal Chemotherapy for Recurrent Mucinous Appendiceal Adenocarcinoma: A Viable Treatment Strategy with Demonstrable Benefit

**DOI:** 10.1245/s10434-023-14422-2

**Published:** 2023-10-23

**Authors:** Neal Bhutiani, Travis E. Grotz, Seth J. Concors, Michael G. White, Beth A. Helmink, Kanwal P. Raghav, Melissa W. Taggart, Karen A. Beaty, Richard E. Royal, Michael J. Overman, Aurelio Matamoros, Christopher P. Scally, Safia Rafeeq, Paul F. Mansfield, Keith F. Fournier

**Affiliations:** 1 Department of Surgical Oncology, The University of Texas MD Anderson Cancer Center, Houston, TX USA; 2 Division of Hepatobiliary and Pancreas Surgery, Department of Surgery, Mayo Clinic, Rochester, MN USA; 3 Department of Colon and Rectal Surgery, The University of Texas MD Anderson Cancer Center, Houston, TX USA; 4 Department of Medical Oncology, The University of Texas MD Anderson Cancer Center, Houston, TX USA; 5 Department of Pathology, The University of Texas MD Anderson Cancer Center, Houston, TX USA; 6 Division of Surgical Oncology, Department of Surgery, Maine Medical Center, Portland, ME USA; 7 Department of Radiology, The University of Texas MD Anderson Cancer Center, Houston, TX USA

**Keywords:** Appendiceal cancer, Recurrence, HIPEC, Cytoreduction

## Abstract

**Introduction:**

Many patients with mucinous appendiceal adenocarcinoma experience peritoneal recurrence despite complete cytoreductive surgery (CRS) and hyperthermic intraperitoneal chemotherapy (HIPEC). Prior work has demonstrated that repeat CRS/HIPEC can prolong survival in select patients. We sought to validate these findings using outcomes from a high-volume center.

**Patients and Methods:**

Patients with mucinous appendiceal adenocarcinoma who underwent CRS/HIPEC at MD Anderson Cancer Center between 2004 and 2021 were stratified by whether they underwent CRS/HIPEC for recurrent disease or as part of initial treatment. Only patients who underwent complete CRS/HIPEC were included. Initial and recurrent groups were compared.

**Results:**

Of 437 CRS/HIPECs performed for mucinous appendiceal adenocarcinoma, 50 (11.4%) were for recurrent disease. Patients who underwent CRS/HIPEC for recurrent disease were more often treated with an oxaliplatin or cisplatin perfusion (35%/44% recurrent vs. 4%/1% initial, *p* < 0.001), had a longer operative time (median 629 min recurrent vs. 511 min initial, *p* = 0.002), and had a lower median length of stay (10 days repeat vs. 13 days initial, *p* < 0.001). Thirty-day complication and 90-day mortality rates did not differ between groups. Both cohorts enjoyed comparable recurrence free survival (*p* = 0.82). Compared with patients with recurrence treated with systemic chemotherapy alone, this select cohort of patients undergoing repeat CRS/HIPEC enjoyed better overall survival (*p* < 0.001).

**Conclusions:**

In appropriately selected patients with recurrent appendiceal mucinous adenocarcinoma, CRS/HIPEC can provide survival benefit equivalent to primary CRS/HIPEC and that may be superior to that conferred by systemic therapy alone in select patients. These patients should receive care at a high-volume center in the context of a multidisciplinary team.

**Supplementary Information:**

The online version contains supplementary material available at 10.1245/s10434-023-14422-2.

For patients with mucinous appendiceal adenocarcinoma, complete cytoreductive surgery (CRS) and hyperthermic intraperitoneal chemotherapy (HIPEC) confers a long-term survival benefit.^1^ Still, despite complete CRS/HIPEC, approximately 20–40% of patients with mucinous appendiceal adenocarcinoma will develop peritoneal recurrence.^2,3^ Recurrence can result from several factors, including missed disease at the time of surgery, persistent disease owing to failure of hyperthermic chemotherapy to effectively eliminate microscopic disease, or both. In general, among patients with recurrent disease, several institutions have advocated for repeat CRS/HIPEC as the ideal treatment.^4–13^

Determining optimal treatment of patients with recurrent mucinous appendiceal adenocarcinoma likely includes an assessment of patterns of recurrence following complete CRS/HIPEC. Previous work among patients with low-grade mucinous appendiceal adenocarcinoma undergoing CRS and early postoperative intraperitoneal chemotherapy noted a predisposition for recurrence in the retroperitoneal, small bowel, and abdominal wall incision.^14^ Identifying strategies to prevent recurrences in these and other high-propensity areas will likely prove critical in both preventing recurrences and designing treatment strategies for patients with recurrent disease.

As the incidence of appendiceal adenocarcinoma increases and more patients undergo repeat CRS/HIPEC, the incidence of recurrent cancer will become increasingly common. Several groups have demonstrated that repeat CRS/HIPEC can safely and effectively be used in the treatment of patients with recurrent mucinous appendiceal adenocarcinoma.^4,7,8,10–13,15^ However, optimizing patient selection for repeat CRS/HIPEC and determining the best treatment strategy for these patients remain unclear. We aimed to augment this data with our institution’s experience, assess patterns of recurrence, and delineate the relative benefit of repeat CRS/HIPEC compared with systemic chemotherapy among patients with recurrent mucinous appendiceal adenocarcinoma.

## Patients and Methods

This study and the database utilized were approved by the University of Texas MD Anderson Cancer Center Institutional Review Board. Patients with appendiceal mucinous adenocarcinoma who underwent complete CRS/HIPEC at the University of Texas MD Anderson Cancer between 2004 and 2021 were identified from a prospectively maintained database. Patients treated for low-grade appendiceal mucinous neoplasm (LAMN) and high-grade appendiceal mucinous neoplasm (HAMN) were excluded. Patients were then stratified with respect to whether they received CRS/HIPEC for recurrent disease or as part of their initial treatment. Patients were excluded if they received prior CRS/HIPEC at an outside facility, or did not have tumor grade, peritoneal cancer index (PCI) score, or completeness of cytoreduction (CC) score captured in our database. Please see Supplementary Fig. 1 for a graphical representation of the filtering strategy applied and the study cohort.

CRS/HIPEC was performed in all patients using the technique previously described by our institution.^16^ All operations were performed by one of three surgeons specializing in peritoneal surface malignancy at our institution. Perfusion usually comprised mitomycin C (25 mg/m^2^ × 90 min), cisplatin (200 mg/m^2^ × 60 min), or oxaliplatin (200 mg/m^2^ × 60 min).

Regarding recurrence evaluation, areas of recurrence were determined based on routine surveillance imaging. Recurrences were considered multifocal if tumor involved two noncontiguous regions or more than two contiguous regions.

A separate cohort of patients with recurrent appendiceal mucinous adenocarcinoma treated with chemotherapy alone who underwent initial CRS/HIPEC at MD Anderson Cancer Center was identified for comparison with patients undergoing repeat CRS/HIPEC for recurrent appendiceal mucinous adenocarcinoma.

Cohorts were compared along demographic, clinicopathologic, perioperative, and survival parameters. Complications were only captured as a binary variable encompassing all Clavien–Dindo grades. Continuous variables were compared using Mann–Whitney *U* test. Categorical variables were compared using chi-squared test or Fisher’s exact test as appropriate. Survival analyses were performed using the Kaplan–Meier method. Multivariable survival analysis was performed using Cox proportion hazards regression. For all analyses, *p* < 0.05 was considered significant. All statistical analyses were performed using MedCalc software (MedCalc, Inc. Ostend, Belgium).

## Results

A total of 529 patients with mucinous appendiceal adenocarcinoma were captured by our internal database who received treatment at MD Anderson Cancer Center between 2004 and 2021. These patients underwent a total of 618 CRS/HIPEC, of which 533 CRS/HIPEC were performed at MD Anderson Cancer Center, while 85 were performed at outside facilities prior to referral. After applying selection criteria, we identified a total of 437 CRS/HIPEC performed for appendiceal adenocarcinoma at MD Anderson Cancer Center, 387 (88.6%) of which were part of initial management, while 50 (11.4%) were for recurrent disease. A flowchart demonstrating patient selection is shown in Supplementary Fig. 1.

Regarding demographic and tumor characteristics, patients undergoing CRS/HIPEC as part of initial treatment had higher body mass index than those undergoing CRS/HIPEC for recurrent disease (27.0 kg/m^2^ initial vs. 24.6 kg/m^2^ recurrent, *p* = 0.01). The groups did not differ with respect to age, gender, tumor grade, PCI score, or receipt of preoperative chemotherapy (Table 1).

**Table 1:** Patient demographics and tumor characteristics

	Initial treatment (*n* = 387)	Recurrent disease (*n* = 50)	*p* value
Age (years) (median, IQR)	55 (46–61)	53 (44–61)	0.54
Female gender (*n*, %)	251 (65%)	38 (76%)	0.15
Body mass index (kg/m^2^) (median, IQR)	27.0 (23.4–31.8)	24.6 (22.0–28.4)	**0.01**
Grade (*n*, %)			
Well differentiated	225 (58%)	33 (66%)	0.36
Moderately differentiated	93 (24%)	12 (24%)	
Poorly differentiated	69 (18%)	5 (10%)	
Signet ring cell (*n*, %)	19 (5%)	0 (0%)	0.25
PCI (median, IQR)	17 (15–19)	18 (12–21)	0.39
Preoperative chemotherapy (*n*, %)	72 (19%)	14 (28%)	0.13

Bold indicates statistically significant finding

Regarding perioperative details, choice of chemotherapy agent for HIPEC differed between the groups. Patients undergoing CRS/HIPEC as part of initial treatment more often underwent perfusion with mitomycin C than those undergoing CRS/HIPEC for recurrent disease (94% initial vs. 20% recurrent), while those undergoing CRS/HIPEC for recurrent disease underwent perfusion with oxaliplatin or cisplatin more often than their initial CRS/HIPEC counterparts (35%/44% recurrent vs. 4%/1% initial, *p* < 0.001) (Table 2). Additionally, operative time for CRS/HIPEC for recurrent disease was longer than for initial disease (median 511 min initial vs. median 629 min recurrent, *p* = 0.002). However, length of stay was shorter among those undergoing CRS/HIPEC for recurrent disease than those undergoing CRS/HIPEC as part of initial treatment (median 10 days recurrent vs. 13 days recurrent, *p* < 0.001). Thirty-day complication (approximately 70%) and 90-day mortality rates (< 1%) did not differ significantly between the two groups.

**Table 2:** Perioperative details

	Initial treatment (*n* = 387)	Recurrent disease (*n* = 50)	*p* value
HIPEC agent (*n*, %)			
Mitomycin C	365 (94%)	10 (20%)	**< 0.001**
Cisplatin	3 (1%)	23 (46%)	
Oxaliplatin	16 (4%)	17 (34%)	
Other	3 (1%)	0 (0%)	
Completeness of cytoreduction (*n*, %)			
0	252 (65%)	28 (56%)	0.63
1	100 (26%)	19 (38%)	
2	24 (6%)	2 (4%)	
3	11 (3%)	1 (2%)	
Operative time (min) (median, IQR)	511 (418–643)	629 (475–746)	**0.002**
Length of stay (days) (median, IQR)	13 (10–19)	10 (8–12)	**< 0.001**
30-Day complications (all grades) (*n*, %)	282 (73%)	33 (66%)	0.32
90-Day mortality (*n*, %)	2 (0.5%)	0 (0%)	1.00

Bold indicates statistically significant finding

**Fig. 1:**
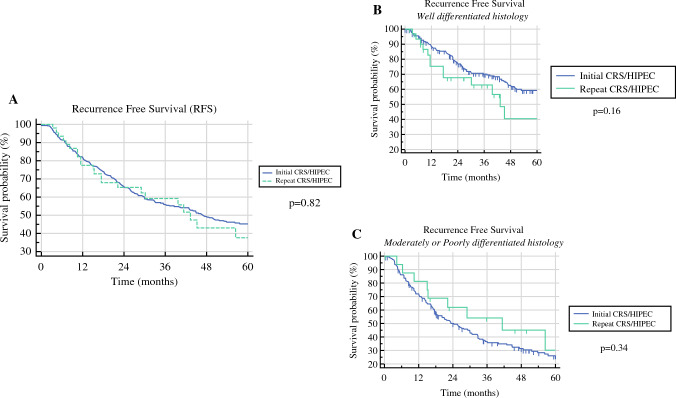
Comparison of recurrence-free survival between initial CRS/HIPEC and repeat CRS/HIPEC among the entire cohort (**A**), among patients with well-differentiated histology (**B**), and among patients with moderately/poorly differentiated histology (**C**)

With respect to follow-up and recurrence, median time to last follow-up was longer for patients undergoing CRS/HIPEC as part of initial treatment compared with those undergoing CRS/HIPEC for recurrent disease (51 months initial vs. 35 months recurrent, *p* = 0.01) (Table 3). Among patients undergoing CRS/HIPEC as part of initial treatment, recurrences most often occurred in the peritoneum (21.3%), pelvis (16.7%), incision/abdominal wall/umbilicus (8.3%), or were multifocal in nature (22.4%) (Table 4). Many of the multifocal recurrences involved the small bowel (18/43, 41.8%). Median time between identification of recurrence and repeat CRS/HIPEC for patients well-differentiated histology was 14.4 months versus 6.5 months for patients with moderately to poorly differentiated histology (*p* = 0.02). Median time between initial operation and repeat CRS/HIPEC for patients with well-differentiated histology was 30.8 months versus 30.0 months for patients with moderately to poorly differentiated histology (*p* = 0.31). Survival analysis comparing recurrence-free survival between patients undergoing CRS/HIPEC as part of initial treatment with those undergoing CRS/HIPEC for recurrent disease demonstrated no difference in recurrence-free survival between the two groups (*p* = 0.82) (Fig. 1a). When analyzing recurrence-free survival for well-differentiated histology and moderately/poorly differentiated histology, respectively, no significant differences were noted between patients undergoing initial and repeat CRS/HIPEC (Fig. 1b, c). Multivariable analysis demonstrated that, when controlling for age, grade, PCI, and CC score, repeat CRS/HIPEC was not significantly associated with a difference in recurrence free survival compared with initial CRS/HIPEC (HR 1.30, 95% confidence interval 0.83–2.03, *p* = 0.25).

**Table 3:** Follow-up and recurrence details

	Initial treatment (*n* = 387)	Recurrent disease (*n* = 50)	*p* value
Time to last follow-up (months; median, IQR)	51 (26–90)	35 (19–62)	**0.01**
Recurrence (*n*, %)	192 (50%)	28 (56%)	0.45

Bold indicates statistically significant finding

**Table 4:** Locations of recurrence

	Recurrence after initial treatment (*n* = 192)
Location of recurrence (*n*, %)	
Peritoneum	41 (21.3%)
Stomach	1 (0.5%)
Small bowel	3 (1.5%)
Colon	5 (2.6%)
Mesentery	3 (1.5%)
Pelvis	32 (16.7%)
Incision/abdominal wall/umbilicus	16 (8.3%)
Pancreas	1 (0.5%)
Liver	4 (2.1%)
Lymph nodes	4 (2.1%)
Retroperitoneum	1 (0.5%)
Chest	14 (7.3%)
Multifocal	43 (22.4%)
Unknown	24 (12.5%)

**Fig. 2:**
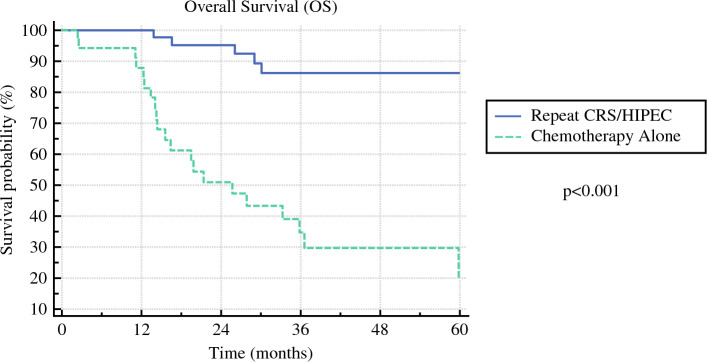
Comparison of overall survival among patients treated with repeat CRS/HIPEC and select patients treated with chemotherapy alone

Finally, a total of 36 patients with appendiceal adenocarcinoma who had undergone initial CRS/HIPEC with CC 0 or 1 at MD Anderson Cancer Center and were treated with chemotherapy alone for their recurrent disease were identified from our database. In comparing patients undergoing CRS/HIPEC for recurrent disease with those with recurrent disease treated with chemotherapy alone, patients treated with chemotherapy alone were older (median age 58 years chemo vs. 53 years CRS/HIPEC, *p* = 0.02) and more likely to have more aggressive histology (75% moderately/poorly differentiated chemo alone vs. 34% moderately/poorly differentiated repeat CRS/HIPEC, *p* < 0.001) (Table 5). Median time to recurrence after initial CRS/HIPEC was 12.0 months for patients treated with chemotherapy alone compared with 21.7 months for patients treated with repeat CRS/HIPEC (*p* = 0.11). Survival analysis demonstrated that repeat CRS/HIPEC was associated with improved overall survival compared with chemotherapy alone (*p* < 0.001) (Fig. 2). On multivariable analysis controlling for age and grade, treatment with chemotherapy alone was independently associated with worse overall survival compared with repeat CRS/HIPEC (HR 8.56, 95% CI 2.95–24.8, *p* < 0.001).

**Table 5:** Cohort comparison of patients treated with repeat CRS/HIPEC versus chemotherapy alone

	Chemotherapy alone (*n* = 36)	Repeat CRS/HIPEC (*n* = 50)	*p* value
Age (years) (median, IQR)	58 (52–62)	53 (33–61)	**0.02**
Female gender (*n*, %)	22 (61%)	38 (63%)	0.16
Grade (*n*, %)			
Well differentiated	9 (21%)	33 (66%)	**< 0.001**
Moderately differentiated	12 (33%)	12 (24%)	
Poorly differentiated	15 (42%)	5 (10%)	

Bold indicates statistically significant finding

As a point of comparison, four patients underwent repeat CRS/HIPEC with incomplete cytoreduction at our institution during the study period. Three of these patients had well-differentiated disease, and one had moderately differentiated disease. Median OS for this cohort was 29.0 months compared with 25.7 months for patients with recurrent disease treated with chemotherapy alone (*p* = 0.71).

Of note, among the five patients with poorly differentiated histology who underwent repeat CRS/HIPEC, none had signet ring cell histology. All patients were treated with preoperative systemic therapy and had either radiographic response or stable disease prior to CRS/HIPEC. Median recurrence-free survival for this cohort was 15.3 months. One patient died from disease, three were alive with disease at last follow-up, and one had no evidence of disease at last follow-up.

## Discussion

Herein, we demonstrate that, among select patients with recurrent mucinous appendiceal adenocarcinoma, repeat CRS/HIPEC represents a safe, effective treatment that provides durable recurrence-free survival benefit. It also offers a significant survival benefit compared with systemic chemotherapy alone. Moreover, we demonstrate that recurrences often occur in the pelvis, peritoneum, and abdominal wall, indicating a potential for targeted strategies at initial CRS/HIPEC to decrease recurrence rates in this patient population.

The findings in our cohort support the current small body of literature describing safety and efficacy of repeat CRS/HIPEC as part of the management for recurrent appendiceal adenocarcinoma. The 0% 90-day mortality, approximately 85% 5-year overall survival, and shorter length of stay compared with initial CRS/HIPEC in our patient cohort reflect those described in recent series and, with respect to survival, represent an improvement from earlier series.^4,7,10–12,15^

However, two points bear mentioning. First, the ability to obtain CC 0 or 1 did not differ between initial CRS/HIPEC and repeat CRS/HIPEC, nor did the median PCI. However, the median operative time was significantly longer by approximately 110 min in the repeat CRS/HIPEC cohort. Broadly speaking, the current literature described mixed data regarding differences in operative time and choosing when to take patients for CRS/HIPEC in the setting of recurrent disease to optimize the ability to achieve CC 0 or 1 cytoreduction.^1,4,6–8,10^ In our experience, after CRS/HIPEC, a subsequent CRS/HIPEC involves 2.5–3 h of adhesiolysis prior to beginning cytoreduction. While they are not more likely to require gastrointestinal diversion (ileostomy, colostomy), they do more often require complex abdominal wall reconstruction. These difference accounts for the additional operative time. Regarding choosing when to operate, we consider, among other parameters, histologic grade, patient functional and nutritional status, symptoms, disease-free interval, and the tumor location/distribution. In general, we would delay a repeat CRS/HIPEC as long as safely feasible. Additionally, we are more likely to offer this to patients with a longer interval from initial CRS/HIPEC to recurrence, a parameter that has been associated with improved outcomes in patients undergoing repeat CRS/HIPEC.^17^ If a patient with a well-differentiated mucinous adenocarcinoma relapses, we often find it early on surveillance imaging and will monitor the patient for symptoms and disease progression. We decide to operate when the progression may increase the complexity of the operation or lead to symptoms. In these patients, we recognize both that the disease is an indolent process and that there are a limited number of times we can repeat the operation safely. As a result, we attempt to extend the interval between CRS/HIPECs, thus extending survival. In contrast, if a patient has a poorly differentiated mucinous adenocarcinoma ± signet ring cells, we typically will treat at earliest recurrence, as the extent of disease is more likely underestimated in this cohort. The role of CRS and HIPEC is less well defined in this cohort taken as a whole, and we currently use response to systemic chemotherapy among other factors for patient selection.

Indeed, the decision regarding which patients are offered repeat CRS/HIPEC with curative intent depends on multiple factors, including many outlined above, particularly patient functional and nutritional status, disease-free interval since last surgery, tumor location/distribution, and the likelihood of achieving a complete cytoreduction. For example, patients with extensive enterocolonic involvement, central mesenteric disease, and/or extensive involvement of the porta hepatis are generally not considered candidates for repeat CRS/HIPEC. Additionally, patients with short-interval recurrence after initial CRS/HIPEC with complete cytoreduction may not have the benefit of repeat CRS/HIPEC outweigh the risks. Indeed, while not statistically significant, the median recurrence free survival in our patients treated with systemic chemotherapy alone for recurrence was 12.0 months, just over half the recurrence-free survival of patients treated with repeat CRS/HIPEC. These patients may be referred to medical oncology for consideration of systemic chemotherapy unless repeat CRS/HIPEC may provide some palliative benefit.

The patterns of recurrence described in the present manuscript bear special mention, particularly with respect to pelvic recurrences. Our experience has been that many of these pelvic recurrences are in women who have had prior gynecological surgery or disease in the ovaries. This observation has caused us to change our practice to include removal of the ovarian vascular pedicle at time of initial CRS/HIPEC and have a low threshold for bilateral oophorectomy if the ovaries appear even minimally involved with disease. Indeed, we have altered our operative technique and patterns on the basis of our observed recurrence patterns. In addition to our approach to the pelvis described above, we perform a systematic peritoneal evaluation, making every effort to achieve a CCR0/1 whenever possible. We carefully plan incisions for diagnostic laparoscopy in the midline, make every effort to avoid off-midline incisions, and strongly consider taking the umbilicus at the time of CRS/HIPEC in an effort to prevent abdominal wall recurrences. Having noted incisional recurrences in these patients, we have adopted the procedure of creating a subcutaneous flap along the fascia prior to closing the abdomen for perfusion. This allows for chemotherapy to perfuse the subcutaneous tissues around the incision. Outcomes of adoption of this technique will be a part of future study.

Among patients with recurrent appendiceal adenocarcinoma, optimal utilization of perioperative chemotherapy and the choice of chemotherapy agent during CRS/HIPEC remain areas without clear consensus. While other centers have described using the same chemotherapy agent, often mitomycin C, for perfusion during repeat CRS/HIPEC, and a recent study has noted no significant difference in outcomes with changing chemotherapy agents, our practice has historically involved changing chemotherapy agents to a platinum-based agent.^9,15,18^ Importantly, as well, previously published data have demonstrated improved overall and recurrence-free survival among patients with a core temperature of at least 38.0 °C for at least 30 min. Thus, we perfuse for at least 60 min with said agent in an effort to achieve this metric.^19^Our rationale for switching agents derives from concern regarding resistance of recurrent disease to the initial chemotherapy agent used during CRS/HIPEC (often mitomycin C). Mitomycin C is not an active chemotherapeutic agent de novo, but requires reductive activation via upregulated enzymes (e.g., DT-diaphorase or NQ01) in tumor cells.^20,21^ Most gastrointestinal malignancies have upregulation of these enzymes and, as a result, are susceptible to mitomycin C. However, in patients with recurrent disease, mitomycin C may have been ineffective during the initial HIPEC owing to low levels of enzyme activity in that the tumor. Thus, we often use a different chemotherapeutic agent in these tumors to avoid administration of mitomycin C to tumor cells that do not metabolize the drug into its toxic metabolites. Additionally, a subset of the present cohort received systemic chemotherapy in the preoperative setting. Our multidisciplinary team uses preoperative chemotherapy to assess disease biology in patients with moderately or poorly differentiated disease. Identifying patients with response to systemic therapy or disease stability allows us to carefully select patients in whom the risk of CRS/HIPEC would be outweighed by the oncologic benefit. While we did so historically, we currently do not administer systemic chemotherapy to patients with well-differentiated disease either in the recurrent setting or in the initial setting given the lack of demonstrable survival benefit these patients derive from systemic chemotherapy administration.^22,23^

This study should be interpreted in light of several limitations. The work reported herein represents a single-institution analysis, and the sample size of the cohort described is limited by exclusion of patients who underwent CRS/HIPEC elsewhere. Many patients presenting to MD Anderson for consideration of repeat CRS/HIPEC, like other high-volume centers, have their initial operation done at a different facility, and confirmation of the completeness of peritoneal evaluation at the time of initial operation remains challenging. Moreover, the limited number of repeat CRS/HIPEC included prevented stratification by pathologic grade to assess grade-specific outcomes. Furthermore, we do not have data on patients who were offered repeat CRS/HIPEC but chose to undergo systemic chemotherapy alone, limiting our ability to directly compare repeat CRS/HIPEC and systemic chemotherapy alone in patients felt to be candidates for repeat CRS/HIPEC. In comparing patients with recurrent disease treated with chemotherapy alone with those treated with repeat CRS/HIPEC, we acknowledge that these groups were inherently different, particularly with respect to tumor differentiation. While these differences render comparison of these groups difficult, we attempted to control for these differences with multivariable analysis. Finally, the lack of Clavien–Dindo grade and the availability of only 30-day rather than 90-day complication rates limits the ability to compare rates of the most clinically impactful (i.e., severe) complications over an extended timeframe that may be more appropriate given the complexity of these cases.

## Conclusions

CRS/HIPEC can confer durable recurrence-free survival benefit equivalent to primary CRS/HIPEC in appropriately selected patients with recurrent appendiceal mucinous adenocarcinoma. Additionally, it may confer improved overall survival in select patients compared with systemic chemotherapy alone. Given the complexity of decision-making surrounding taking patients to the operating room and the technical challenges associated with repeat CRS/HIPEC, patients with recurrent appendiceal mucinous adenocarcinoma should be cared for in high-volume centers in the context of an experienced multidisciplinary team.

### Supplementary Information

Below is the link to the electronic supplementary material.

**Supplementary Fig. 1** Cohort selection methodology
