# Supplementary figures and images for: Repeat Cytoreduction and Hyperthermic Intraperitoneal Chemotherapy for Recurrent Mucinous Appendiceal Adenocarcinoma: A Viable Treatment Strategy with Demonstrable Benefit
Source: Ann Surg Oncol. 2023 Oct 23;31(1):614–21. doi: 10.1245/s10434-023-14422-2 (PMC10695875; doi:10.1245/s10434-023-14422-2)

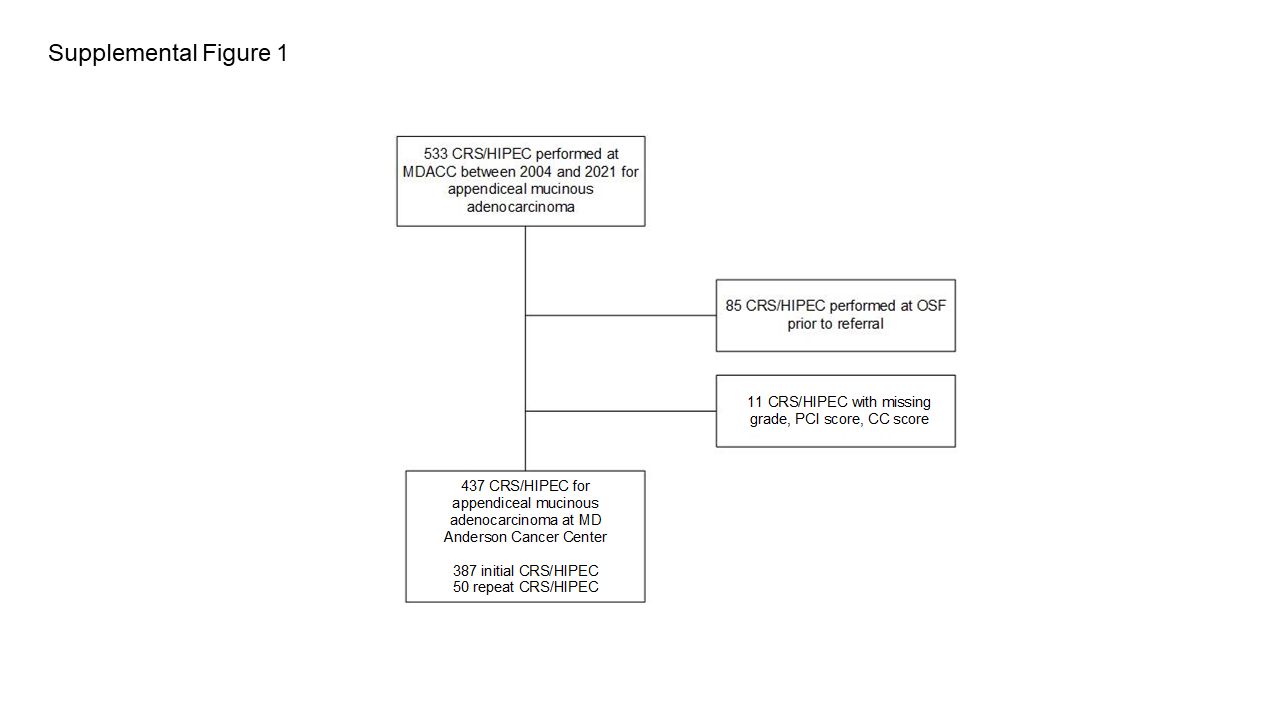

Supplement: Supplementary file 1 — Supplementary Fig. 1 Cohort selection methodology [file 10434_2023_14422_MOESM1_ESM.tif]
